# Time‐dependent and nonlinear effects of prognostic factors in nonmetastatic colorectal cancer

**DOI:** 10.1002/cam4.1116

**Published:** 2017-07-14

**Authors:** Sheng‐Qiang Chi, Yu Tian, Jun Li, Dan‐yang Tong, Xiang‐Xing Kong, Graeme Poston, Ke‐Feng Ding, Jing‐Song Li

**Affiliations:** ^1^ Engineering Research Center of EMR and Intelligent Expert System Ministry of Education Collaborative Innovation Center for Diagnosis and Treatment of Infectious Diseases College of Biomedical Engineering and Instrument Science Zhejiang University No. 38 Zheda Road Hangzhou Zhejiang 310027 China; ^2^ Department of Surgical Oncology Second Affiliated Hospital Zhejiang University School of Medicine No. 88 Jiefang Road Hangzhou 310009 Zhejiang Province China; ^3^ Department of Surgery Aintree University Hospital Liverpool L9 7AL United Kingdom

**Keywords:** Colorectal cancer, nonlinear effects, SEER, time‐dependent effects

## Abstract

The survival risk following curative surgery for nonmetastatic colorectal cancer (CRC) may be over‐ or underestimated due to a lack of attention to nonlinear effects and violation of the proportional hazards assumption. In this paper, we aimed to detect and interpret the shape of time‐dependent and nonlinear effects to improve the predictive performance of models of prognoses in nonmetastatic CRC patients. Data for nonmetastatic CRC patients diagnosed between 2004 and 2012 were obtained from the Surveillance Epidemiology End Results registry. Time‐dependent and nonlinear effects were tested and plotted. A nonlinear model that used random survival forests was implemented. The estimated 5‐year cancer‐specific death rate was 17.95% (95% CI, 17.70–18.20%). Tumor invasion depth, lymph node status, age at diagnosis, tumor grade, histology and tumor site were significantly associated with cancer‐specific death. Nonlinear and time‐dependent effects on survival were detected. Positive lymph node number had a larger effect per unit of measurement at low values than at high values, whereas age at diagnosis showed the opposite pattern. Moreover, nonproportional hazards were detected for all covariates, indicating that the contributions of these risks to survival outcomes decreased over time. The nonlinear model predicted prognoses more accurately (C‐index: 0.7934, 0.7933–0.7934) than did the Fine and Gray model (C‐index: 0.7550, 0.7510–0.7583). The three‐dimensional cumulative incidence curves derived from nonlinear model were used to identify the change points of the risk trends. It would be useful to implement these findings in treatment plans and follow‐up surveillance in nonmetastatic CRC patients.

## Introduction

Colorectal cancer (CRC) is the third most common cancer in men and the second most common cancer in women, contributing to an estimated 1.4 million cases and 693,900 deaths in 2012 around the world 1. In the United States, CRC is not only the third most common cancer but also the third leading cause of cancer deaths 2. In China, CRC is the second leading cause of 5‐year cancer‐specific prevalence 3. It is therefore important to provide clinicians with prognostic information for making treatment decisions. The American Joint Committee on Cancer (AJCC) tumor node metastasis (TNM) staging system is widely used to predict prognoses in CRC patients and to guide adjuvant therapy. However, the TNM treats positive lymph node (PLN) number, a continuous variable, as a categorical variable, which can result in the loss of prognosis information. Published population‐based studies have indicated that the complex modifications proposed in the 7th edition of TNM may fail to address all survival discrepancies 4, 5, 6. Because there is controversy regarding TNM's level of accuracy, many studies have attempted to restage specimens to obtain better predictive performance, primarily using survival analyses for modeling and evaluation.

Conventional survival analysis methods, including the cumulative incidence competing risk (CICR) and Fine and Gray models, are widely applied in cancer prognosis research in the presence of competing risks 7, 8. However, both the CICR and Fine and Gray models have drawbacks. First, in the Fine and Gray model, the proportional hazards assumption (PHA) is usually violated or not tested. As Buchholz and Sauerbrei 9 summarized, time‐dependent effects have been detected for prognostic factors in many cancers; for example, associated with estrogen receptor expression 10 and tumor size 11 in breast cancer and with Karnofsky performance status 12 in ovarian cancer. Second, it is unknown whether the cutoff values based on categories of continuous data are optimal, and the continuous, nonlinear effects of predictors are frequently ignored. Analyses 7, 8 based on Fine and Gray models assume that predictive factors act linearly on the log hazard function, whereas those based on the extended model that attempt to detect and interpret the shape of time‐dependent effects present challenges in predefining a function of time or selecting the correct functional form for a continuous factor 9. Joensuu et al. 13 investigated proportional hazards and nonlinear effects of prognostic factors on the risk of recurrence in gastrointestinal stromal tumors after surgery and found that prognoses were more accurately predicted by the novel nonlinear model than by commonly used risk‐stratification schemes. However, time‐dependent and nonlinear effects of predictors of CRC prognoses remain poorly studied.

In this paper, we attempt to detect and interpret the shape of time‐dependent and nonlinear effects using population‐based CRC survival data. We develop a model to predict CRC outcomes in the presence of competing risks, including the extent of cancer at diagnosis and patient demographic characteristics, as predictors to address time‐dependent and nonlinear effects. Our work is distinct from previous studies because age and PLN number were treated as continuous, nonlinear variables rather than as categorical variables. We used cancer‐specific death (CSD) as the outcome of interest and considered death from other causes as a competing risk. We used a novel machine learning method (random survival forests [RSF] for competing risks 14) with no restrictions and assumptions to build a prediction model.

## Materials and Methods

### Patients

Data on patients who were diagnosed with CRC were obtained from the November 2014 update of the National Cancer Institute SEER 15 including 18 population‐based registries. Patient information, including demographics, diagnoses and survival, are routinely collected, and this information is publicly available as deidentified data.

In this paper, the analysis was limited to patients who were diagnosed with primary nonmetastatic CRC (SEER primary site recodes C180‐C189, C260, C199, or C209 without distant metastasis) as their only malignant tumor and who had undergone potentially curative oncologic resection from January 2004 through December 2012. We excluded individuals if their cancer‐reporting source was a nursing home, hospice, autopsy or death certificate; if their survival time was less than 1 month; if their N stage was N1c; or if they had in situ disease. In addition, patients with unknown key predictor variables were excluded (Fig. S1).

### Statistical analysis

Survival outcomes were determined based on the cutoff time of December 2012. Because predicting prognosis was our goal, we evaluated CSD while treating death of other causes as a competing risk. The study population therefore displayed three different mortality statuses: (1) alive at the cutoff time (2) death from cancer, (3) death from other causes. Survival time was calculated from the date of diagnosis to the cutoff date in surviving patients or to the date of death in all others. The covariates used in the analysis were selected based on clinically relevant factors obtained from the literature and included age at diagnosis, gender, tumor site, histology, tumor grade, N stage, and tumor extension, which is referred to as T stage. Univariate analyses were performed to identify criteria (*P* < 0.05) to include as risk factors in the final multivariate model with the cumulative incidence competing risk method based on the cumulative incidence function (CIF), and Gray's test 16 was used to test for equality in the CIFs across groups. Multivariate modeling (using the first group of each factor as the baseline) in the presence of competing risks was performed with a Fine and Gray model 17. The PHA was tested using Schoenfeld residuals, which were scaled and plotted over time for each covariate.

Nonlinear modeling was performed using an RSF model for competing risks, which is an extension of random forests for right‐censored survival data analyses. The nonparametric RSF model can assess nonlinear effects of variables and explore complex interactions between covariates without a restrictive assumption, whereas the Fine and Gray model is log‐linear, potentially resulting in the loss of nonlinear effects after covariate categorization; RSF can model smooth nonlinear effects.

The partial dependence of predicted CIFs after 1–5 years was plotted to visualize the nonlinear effects of the predictors. A concordance index, which corresponds to the area under a receiver operating characteristic curve, was calculated to measure the predictive accuracy. Bootstrapping, evaluating the mean concordance index and its confidence interval, was used to measure the reliability of the Fine and Gray model. Out‐of‐bag ensembles 14 (cross‐validation) was used to measure the reliability of the RSF for competing risks (detailed in the appendix). All analyses were performed using R version 3.3.0 and R packages. Finally, a web‐based application was developed to use the final model to predict CSD.

## Results

### Patient demographics and tumor characteristics

**Figure 1:**
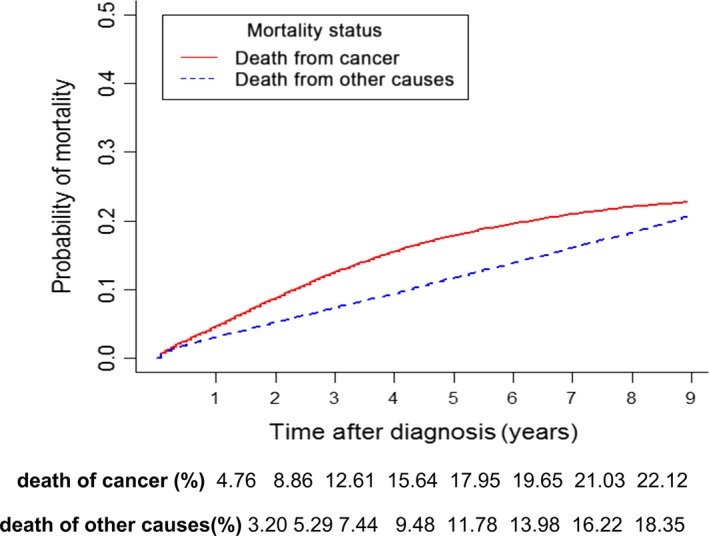
Cumulative incidence curves for cancer‐specific mortality and mortality from other causes, which were evaluated as competing events in patients with nonmetastatic colorectal cancer.

A total of 131,289 patients met the inclusion criteria and were included in the analysis. The patient baseline and tumor characteristics are listed in Table 1. The median follow‐up time was 38 months (range, 1–107 months). Cancer‐specific and other cause‐specific deaths were recorded in 19,301 (14.70%) and 13,719 (10.45%) patients, respectively. The median age at diagnosis was 65 years old, and only 60 (0.05%) patients were younger than 20 years old. Patients were categorized into age groups of <55 years, 55–64 years, 65–74 years, and >74 years. The median PLN number was 0 (interquartile range, 0–2; maximum, 73). The probability of death from cancer was significantly greater than that of death from other causes (Fig. 1). The increasing rate (slope of the cumulative incidence curve) of probability of death from cancer decreased as time processed, whereas the increasing rate of probability of death from other causes remained constant. The estimated 1‐year to 5‐year cumulative incidences of death from cancer and other causes are listed in Figure 1.

**Table 1:** Demographic and clinical characteristics of SEER nonmetastatic CRC patients from 2004–2012

Characteristic	Number (%)	Cancer death (%)	Other death (%)
Age at diagnosis			
<55	28017 (21.340)	3060 (15.854)	573 (4.177)
55–64	29418 (22.407)	3424 (17.740)	1227 (8.944)
65–74	32208 (24.532)	4364 (22.610)	2748 (20.031)
≥75	41646 (31.721)	8453 (43.796)	9171 (66.849)
Gender			
Male	65598 (49.965)	9571 (49.588)	6845 (49.894)
Female	65691 (50.035)	9730 (50.412)	6874 (50.106)
T stage			
T1	17575 (13.386)	659 (3.414)	1507 (10.985)
T2	23102 (17.596)	1542 (7.989)	2721 (19.834)
T3	75910 (57.819)	12390 (64.194)	8210 (59.844)
T4a	8268 (6.298)	2316 (11.999)	780 (5.686)
T4b	6434 (4.901)	2394 (12.404)	501 (3.652)
N stage			
N0	82998 (63.218)	7169 (37.143)	9484 (69.130)
N1a	15480 (11.791)	2655 (13.756)	1515 (11.043)
N1b	15301 (11.654)	3372 (17.471)	1349 (9.833)
N2a	9684 (7.376)	2864 (14.839)	804 (5.860)
N2b	7826 (5.961)	3241 (16.792)	567 (4.133)
Grade			
Well differentiated	12262 (9.340)	1090 (5.647)	1281 (9.337)
Moderately differentiated	94859 (72.252)	12446 (64.484)	9833 (71.674)
Poorly differentiated	21747 (16.564)	5199 (26.936)	2369 (17.268)
Undifferentiated	2421 (1.844)	566 (2.932)	236 (1.720)
Histology			
Nonmucinous adenocarcinoma	116997 (89.114)	16386 (84.897)	12065 (87.944)
Mucinous adenocarcinoma	11748 (8.948)	2187 (11.331)	1439 (10.489)
Signet ring adenocarcinoma	1096 (0.835)	413 (2.14)	107 (0.780)
Epidermoid carcinoma	79 (0.060)	24 (0.124)	6 (0.044)
Sarcoma	24 (0.018)	12 (0.062)	0 (0)
All other types	1345 (1.024)	279 (0.155)	102 (0.743)
Site			
Left colon	59888 (45.615)	9089 (47.091)	7517 (54.793)
Right colon	38980 (29.690)	5253 (27.216)	3709 (27.035)
Rectum	32421 (24.694)	4959 (25.693)	2493 (18.172)

The left colon includes the splenic flexure, descending colon, and sigmoid colon; and the right colon includes the cecum, appendix, ascending colon, hepatic flexure, and transverse colon.

### Univariate analysis

**Figure 2:**
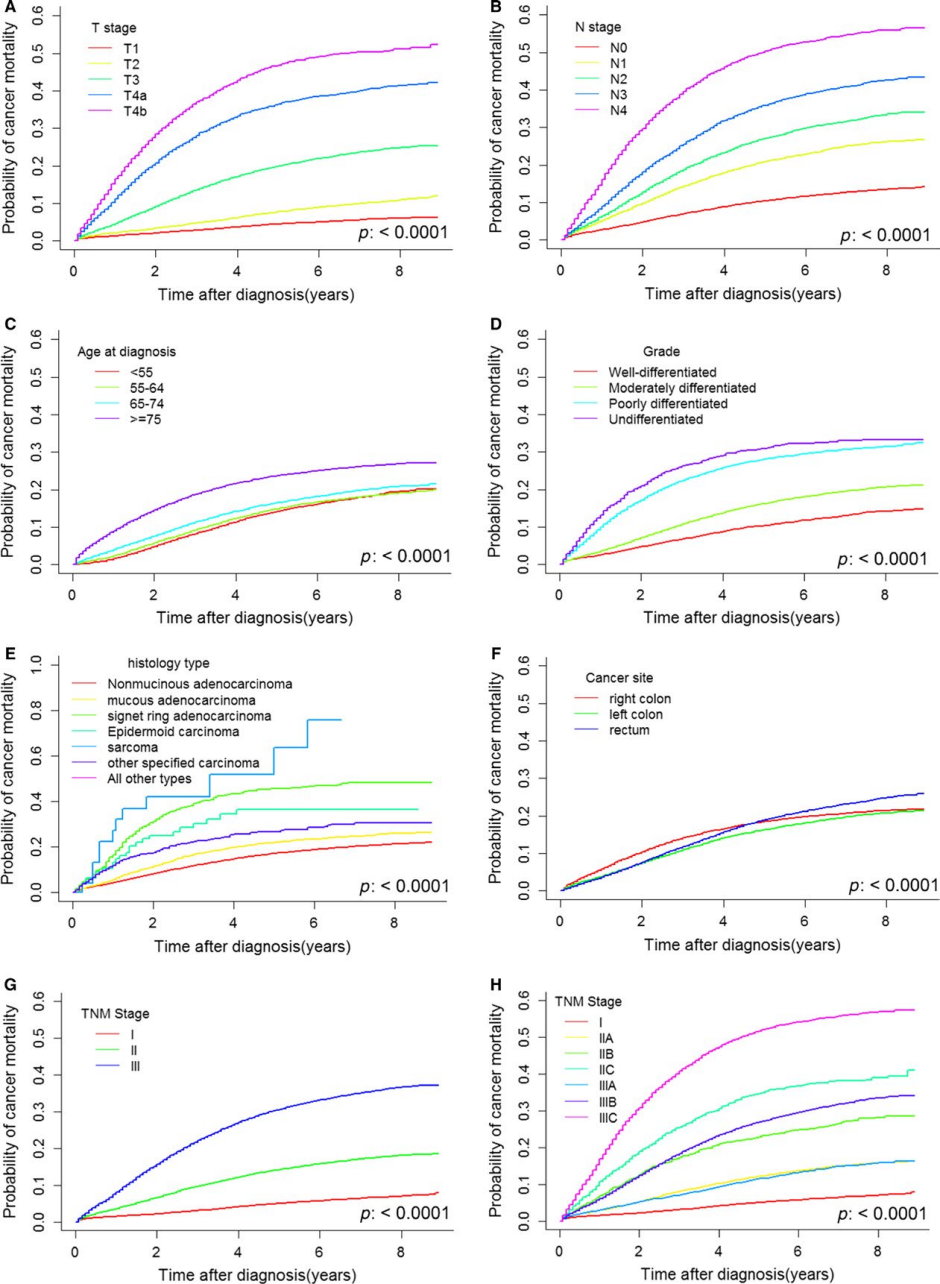
The estimated cumulative incidence curves for T stage (A), N stage (B), age at diagnosis (C), tumor grade (D), histological type (E), tumor site (F), TNM stage (G), and TNM stage subgroups (H) from the univariate analysis performed using the cumulative incidence competing risk method. The left colon includes the splenic flexure, descending colon, and sigmoid colon; and the right colon includes the cecum, appendix, ascending colon, hepatic flexure, and transverse colon. The subgroups of TNM stage were designated according to the 7th edition of the AJCC TNM staging system (stage I: T1‐2N0, stage IIA: T3N0, stage IIB: T4aN0, stage IIC: T4bN0, stage IIIA: T1‐2N1 and T1N2a, stage IIIB: T3‐4aN1, T2‐3N2a, and T1‐2N2b, stage IIIC: T4bN1, T4N2a, and T3‐4N2b).

In the univariate analyses, as expected, we found that T stage, N stage, age, tumor grade, histology, and tumor site were strongly associated with CSD (Fig. 2A–F). There were no gender differences (*P* = 0.275; see Fig. S2 for cumulative incidence curves). The cumulative incidence increased as age increased. Nonmucinous adenocarcinoma was associated with better survival outcomes than were other histological types, and sarcoma had the worst prognosis. Epidermoid carcinoma was associated with better outcomes than was signet ring adenocarcinoma but worse outcomes than was mucinous adenocarcinoma. Worse outcomes were associated with right colon and rectal cancer than with left colon cancer. Greater tumor invasion depth, higher N stage, and worse tumor cell differentiation were associated with poorer survival outcomes. We also drew curves for TNM stages I, II, and III (Fig. 2G) and subgroups of stages II and III (Fig. 2H). The CSD of stage IIIA patients was the same as that of stage IIA patients. In the first 2 years, the CSD of stage IIB patients was almost the same as that of stage IIIB patients; thereafter, the prognosis of stage IIIB patients became poorer than that of stage IIB patients.

### Time‐dependent effects

**Figure 3:**
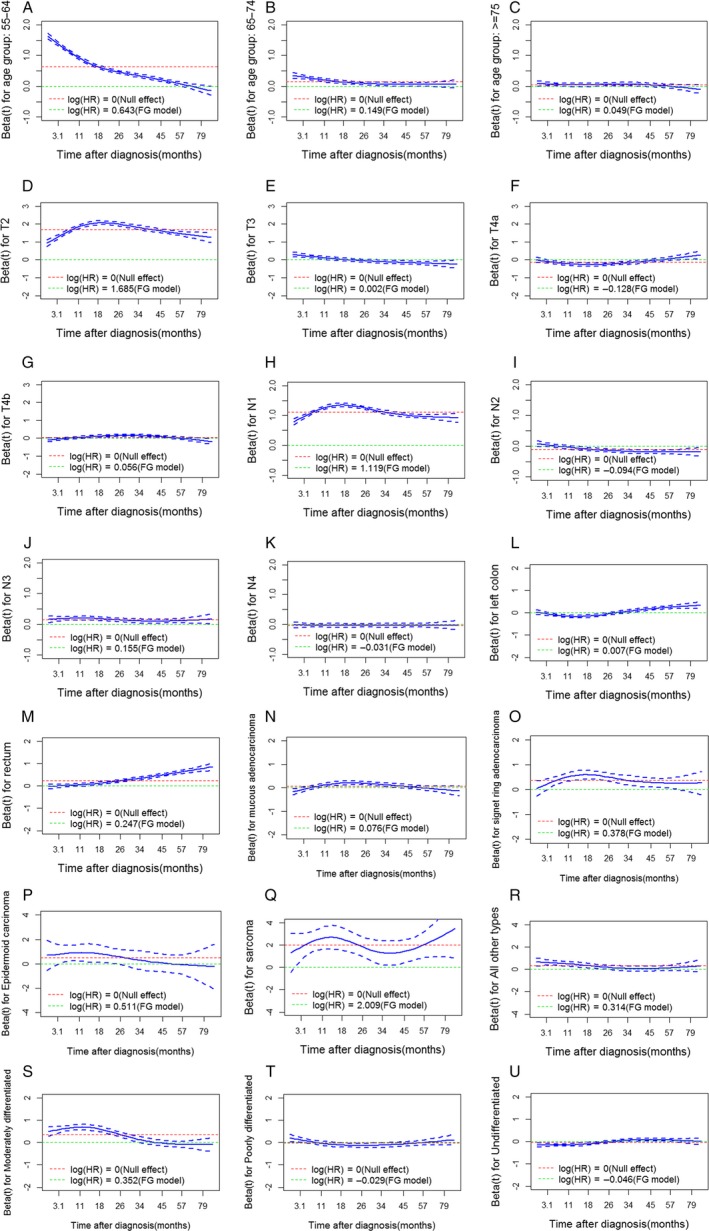
Scaled Schoenfeld residuals for age at diagnosis, T stage, N stage, tumor site, histological type, and tumor grade with 95% confidence intervals. Residuals were used to visualize the log cause‐specific hazard rates for each covariate over time. Green lines represent the null effect (no effect on survival outcomes when Log(HR) is equal to 0), and red lines represent the average log cause‐specific hazard rate as estimated using the Fine and Gray (FG) model. The 55–64‐year‐old age group (A), 65–74‐year‐old age group (B), T3 (E), T4a (F), N1a (H), N1b (I), moderately differentiated tumor grade (S), undifferentiated tumor grade (U), other histological types (R), and tumor site (L, M) were found to be most likely to contribute to nonproportionality. For example, for age between 55 and 64 years, the effect changed over time, tending to diminish in the early years and then become protective later. For age between 65 and 74 years, this group had a higher risk than did the baseline group (age < 55 years) in the early years, whereas this impact tended to disappear later. For age older than 74 years, the effect tended to be constant. The FG model in the figure represents the Fine and Gray model, and HR represents the subdistribution hazard rate.

Based on the univariate analysis results, we used T stage, N stage, age at diagnosis, tumor grade, histology, and tumor site as predictive factors in a multivariate Fine and Gray regression model. The PHA was tested, and the corresponding *P*‐values and the *P*‐value associated with a global test of nonproportionality are reported (Table 2). Scaled Schoenfeld residuals were plotted over time for each covariate (Fig. 3). The results of the global test suggested strong evidence of nonproportionality (*P* < 0.0001). All covariates were likely to violate the PHA and had time‐dependent effects on the CIF. For example, for age between 55 and 64 years, the effect (Log (HR)) changed over time, tending to diminish in the early years and then become protective (Log (HR) < 0). For the age group of 65–74 years, the risk was higher than that of the baseline group (age <55) in the early years, but then the effect tended to disappear. For age over 74, the effect tended to be constant (Log (HR) changed little).

**Table 2:** Results of the fine and gray model and tests of the proportional hazard assumption**.**

Covariate	Coefficient	Standard Error	*P* > |*z*|	HR	Lower 0.95	Upper 0.95	p(PH)
Age: 55–64	0.643	0.016	<0.0001	1.902	1.845	1.961	<0.0001
Age: 65–74	0.149	0.016	<0.0001	1.161	1.126	1.197	<0.0001
Age: ≥75	0.049	0.016	0.002	1.050	1.018	1.084	0.144
T2	1.685	0.031	<0.0001	5.393	5.075	5.731	0.386
T3	0.002	0.026	0.923	1.003	0.953	1.054	<0.0001
T4a	−0.128	0.025	<0.0001	0.880	0.837	0.924	0.009
T4b	0.056	0.018	0.002	1.058	1.022	1.095	0.544
N1a	1.119	0.017	<0.0001	3.063	2.964	3.166	0.039
N1b	−0.094	0.016	<0.0001	0.910	0.881	0.940	<0.0001
N2a	0.155	0.018	<0.0001	1.167	1.126	1.210	0.148
N2b	−0.031	0.018	0.081	0.969	0.935	1.004	0.984
Moderately differentiated	0.352	0.036	<0.0001	1.422	1.325	1.525	<0.0001
Poorly differentiated	−0.029	0.028	0.301	0.972	0.921	1.026	0.198
Undifferentiated	−0.046	0.016	0.005	0.956	0.926	0.986	<0.0001
Mucous adenocarcinoma	0.076	0.023	0.00101	1.079	1.031	1.129	0.981
Signet ring adenocarcinoma	0.378	0.051	<0.0001	1.459	1.320	1.614	0.743
Epidermoid carcinoma	0.511	0.205	0.013	1.667	1.116	2.490	0.095
Sarcoma	2.009	0.290	<0.0001	7.454	4.220	13.166	0.901
All other types	0.314	0.061	<0.0001	1.369	1.214	1.544	0.003
Left colon	0.007	0.018	0.704	1.007	0.972	1.042	<0.0001
Rectum	0.247	0.018	<0.0001	1.280	1.235	1.327	<0.0001

Proportional hazard assumption test: GLOBAL < 0.0001.

**Figure 4:**
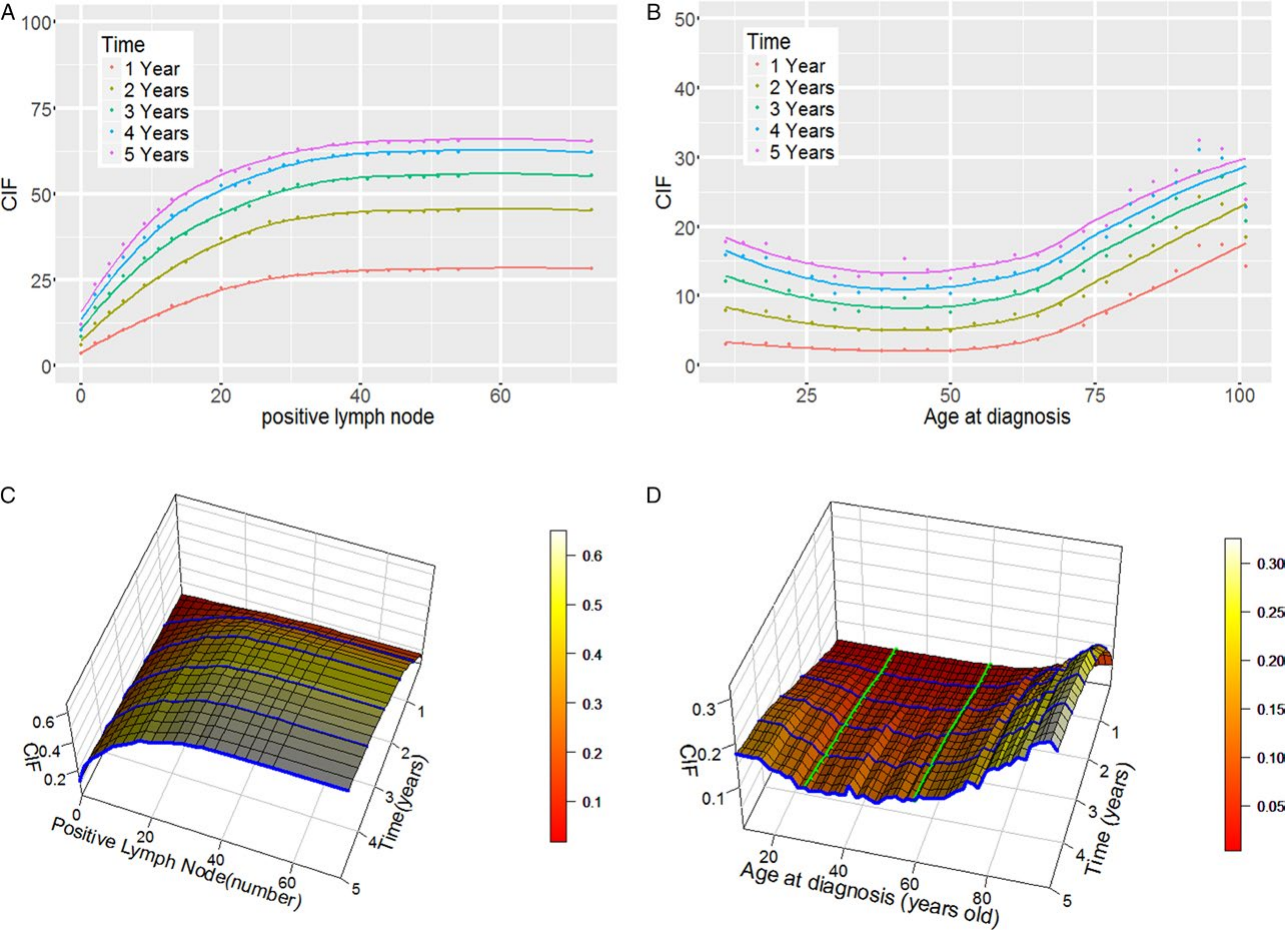
Nonlinear effects of positive lymph node and age at diagnosis in nonmetastatic colorectal cancer and their three‐dimensional cumulative incidence curves. Panel A shows the nonlinear effect of a diagnosis of positive lymph nodes; panel B shows the nonlinear effect of age at diagnosis; panel C shows the cumulative incidence function of a diagnosis of positive lymph nodes, where the blue lines represent 1‐ to 5‐year cumulative incidence functions as the number of positive lymph nodes increases; and panel D shows the cumulative incidence function of age at diagnosis, where the blue lines represent 1–5‐year cumulative incidence functions as age at diagnosis increases and the green lines represent cumulative incidence functions as time progresses when age at diagnosis was fixed at 30 and 60 years old. CIF represents the cumulative incidence function.

Because many covariates had a nonconstant subdistribution hazard ratio, it was difficult to fit all time‐dependent covariates with a manually determined time function. In addition, the effect of age at diagnosis and lymph node status was evaluated using log‐linear modeling with a proportional hazard assumption, which is typically violated in long‐term follow‐up survival analyses 18, 19. However, we found that age at diagnosis and PLN number had nonlinear effects on survival. We applied RSF to fit the complex time‐dependent and nonlinear effects in the presence of competing risks, using T stage, tumor grade, tumor site, histology, age, and PLN number as covariates. Age and PLN number were treated as continuous nonlinear covariates. The nonlinear effects and CIFs of age and PLN number (evaluated as continuous variables) are shown in Figure 4.

### Nonlinear effects

The strong nonlinear effects of PLN number and age at diagnosis were clearly revealed because the trends of CIF change with PLN number and age at diagnosis were not linear (Fig. 4A and B). We determined that these nonlinear predictors could be transformed to linear effects by separating the predictor values into different bins. The effect of PLN number on CIF was linear at values <15, >30 and between 15 and 30. The effect of age at diagnosis was linear in patients younger than 30 or older than 60. All patients with more than 30 PLNs had almost the same risk, whereas patients who had fewer than 30 PLNs had increased risk as the number of PLNs increased. Age at diagnosis was not associated with risk in patients 30–60 years old, whereas risk decreased with age in patients who were younger than 30, and risk sharply increased with age in patients older than 60. These patterns might explain why studies that treated PLN number and age as categorical rather than continuous variables have often detected no linear effects; in these cases, categorization might have resulted in a loss of prognostic information as described below.

To comprehensively display the relationships between the continuous variables and their corresponding CIF values, we plotted three‐dimensional cumulative incidence curves (Fig. 4C and D). As the number of PLNs increased, survival outcomes worsened regardless of whether the patient was at the 1‐year, 2‐year, 3‐year, 4‐year, or 5‐year time point. CIF increased in patients with a certain number of PLNs as time progressed. In relation to age at diagnosis, the CIF of a particular patient may increase with time. However, when CIF was analyzed across patients at the same time point after diagnosis, the trend in outcomes differed depending on whether the patients were younger than 30, older than 60, or between 30 and 60 years old. By evaluating the three‐dimensional curves, we readily identified the change points of the risk trend and the cumulative incidence curve for each value of the covariate.

### Model evaluation and application

The predictive accuracy of the nonlinear and Fine and Gray models were compared using the concordance index. The nonlinear model was a better predictor of cancer‐specific death in the presence of competing risks in patients with nonmetastatic CRC after surgery. The concordance index value was higher for the nonlinear model (0.7934, 95% CI: 0.7933–0.7934) than for the Fine and Gray model (0.7550, 95% CI: 0.7510–0.7583; *P* < 0.0001; Fig. S3).

The web‐based application developed in this study is available for desktop computers and mobile devices and was implemented to achieve survival outcome predictions (the interface is shown in Fig. S4).

## Discussion

In this study, we used population‐based data from the SEER database to detect and interpret the shapes of time‐dependent and nonlinear effects of clinico‐pathological and demographic characteristics, which can be used to predict CRC prognoses. The study period was between 2004 and 2012, an era in which the patients benefited from modern therapies with improved survival probabilities 20. A new population‐based analysis was needed to determine CRC survival probabilities, as older models 21, 22 are based on data from an earlier period and might be less effective. Strong nonlinear relationships were identified between CIFs and predictors and between CIFs and the nonproportional hazards of predictors. To the best of our knowledge, this study is the first to explore the time‐dependent and nonlinear effects of predictors of CRC survival outcomes in the presence of competing risks with a population‐based dataset. The model that considered time‐dependent and nonlinear effects achieved more accurate predictions than those achieved using models that ignored them to estimate CIFs in patients diagnosed with primary nonmetastatic CRC as their only malignant tumor.

Nonlinear and time‐dependent effects on survival outcomes were detected. Many previous studies have ignored these effects. The widely used 7^th^ AJCC TNM categorizes PLN number and ignores its nonlinear effects, potentially resulting in abrupt changes in estimated CIFs when PLN number approaches a cutoff value. Moreover, differences between patients with more than seven PLNs are generally ignored, and age at diagnosis is typically not taken into account. Our nonlinear model regards T stage, tumor grade, histology, tumor site, PLN number, and age at diagnosis as predictors. The latter two of these factors were viewed as continuous and nonlinear variables, and we observed that both PLN number and age at diagnosis had nonlinear effects on CIFs. PLN number had a larger effect per unit of measurement at low values than at high values, whereas age at diagnosis showed the opposite trend. Evidence for nonproportional hazards was also detected, indicating that all of the variables had time‐dependent impacts on the CIFs. The reasons for these effects should be studied further. However, many previous studies that have analyzed survival using a Cox model or Fine and Gray model, which rely on the PHA, did not test whether their data met this assumption 23. As these models assume proportional hazards, their use may have resulted in the over‐ or underestimation of survival hazards in previous studies. We achieved better performance using a model (RSF) that considered nonproportional hazards and nonlinearity than we did using a Fine and Gray model.

By producing three‐dimensional curves, we readily obtained the change points of the risk trend and the cumulative incidence curve for each value of the covariate. Age is an important demographic factor that is associated with prognosis and has been widely studied. Studies have shown that survival outcomes differ between young and old patients 24, 25. However, our study found that patients younger than 30 years old appeared to have worse outcomes than did patients aged between 30 and 60 years old and that CIFs decreased as age increased. In this study, the number of patients younger than 30 years old was small because of the rarity of this cancer in this age group (766/131,289 or 0.65%). Therefore, the results for these patients should not be used to guide the treatment decision‐making process. However, because there has been a significant increase in the incidence of CRC in young adults 26, studies are needed to determine the prognosis for young CRC patients. The CIFs increase at a slower rate when the number of PLNs is between 15 and 30 than when the number is fewer than 15, and the CIFs appear to remain stable at a PLN number >30.

We constructed a web‐based application to obtain prognosis predictions to facilitate the use of the complex model that was developed in this study. This predictive model can be accessed using a personal computer or mobile smartphone, which may help clinicians counsel patients. Because it is derived from data from a large number of patients and due to the standardized collection and formatting of data, a SEER database‐based prognostic model can be generalized to a wide range of CRC patients and be used to predict prognoses.

However, the SEER data do not include therapeutic and molecular data that might further improve the predictive accuracy of the model, according to recent studies 27, 28. Therefore, we will perform additional studies that combine demographic and clinico‐pathological data with treatment and molecular data to more accurately illustrate the development of CRC and predict prognoses. Moreover, patients with N1c were excluded from this study because the diagnostic criteria of N1c (representing tumor deposits) were not constant but changed several times in the recent versions of the TNM staging systems 29. However, tumor deposits do have an impact on CRC prognoses 30 and should be further studied. Another limitation of this study was that the longest follow‐up in the SEER database was only 107 months, and the median follow‐up was 38 months, which is short for a population with a potentially curative condition. Therefore, the results should be verified by other databases with long‐term follow‐up data.

We conclude that T stage, N stage (or PLN number), age at diagnosis, tumor grade, histology, and tumor site play significant roles in nonmetastatic CRC prognoses. All these covariates have time‐dependent effects on survival outcomes, indicating that the Fine and Gray model may be not suitable for use in CRC survival analyses in the presence of competing risks. The TNM categorization of continuous variables as categorical ones may decrease their predictive accuracy and result in the loss of prognostic information. Models that consider both the continuous and the nonlinear effects of PLN number and age at diagnosis, along with nonproportional hazards, are likely to produce more accurate estimations of survival outcomes.

## Conflict of Interest

None declared.

## Supporting information


**Figure S1.** The details of the data screening procedure.
**Figure S2.** The Estimated cumulative incidence curves by gender from univariate analysis with cumulative incidence competing risk method.
**Figure S3.** Predictive accuracy for the Fine and Gray model and the random survival forests.
**Figure S4.** Interactive web‐based application interface for predicting prognoses.
**Data S1.** Methods.
