# Supplementary material for: Time‐dependent and nonlinear effects of prognostic factors in nonmetastatic colorectal cancer
Source: Cancer Med. 2017 Jul 14;6(8):1882–92. doi: 10.1002/cam4.1116 (PMC5548888; doi:10.1002/cam4.1116)
Supplement: Supplementary file 1 — Figure S1. The details of the data screening procedure. Figure S2. The Estimated cumulative incidence curves by gender from univariate analysis with cumulative incidence competing risk method. Figure S3. Predictive accuracy for the Fine and Gray model and the random survival forests. Figure S4. Interactive web‐based application interface for predicting prognoses. Data S1. Methods. [file CAM4-6-1882-s001.docx]

# Supplementary Material

FIGURES

**Fig. S1 The details of the data screening procedure.**

**Fig. S2 The Estimated cumulative incidence curves by gender from univariate analysis with cumulative incidence competing risk method.**


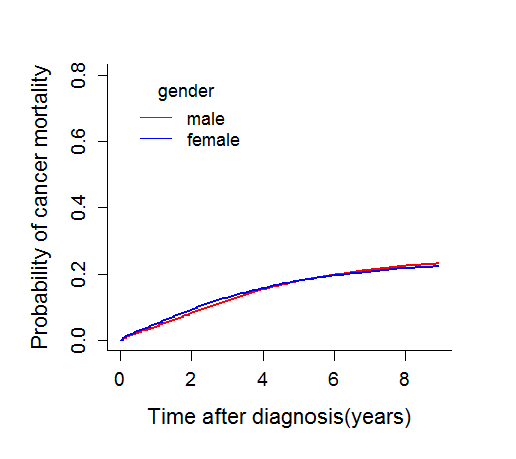


**Fig. S3 Predictive accuracy for the Fine and Gray model and the random survival forests.** RSF represents the random survival forests.

**
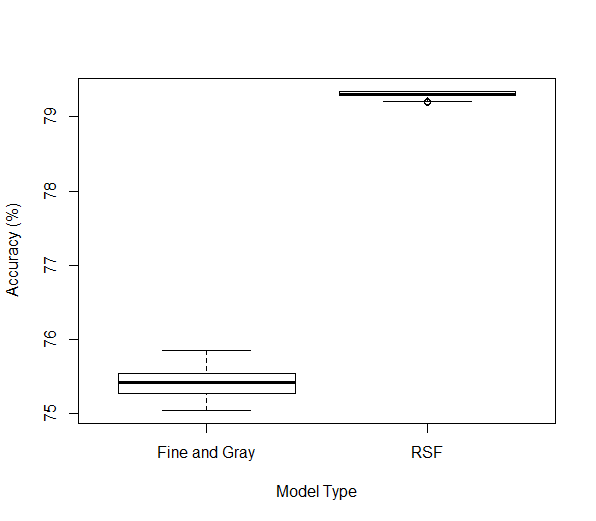
**

**Fig. S4 Interactive web-based application interface for predicting prognoses.**


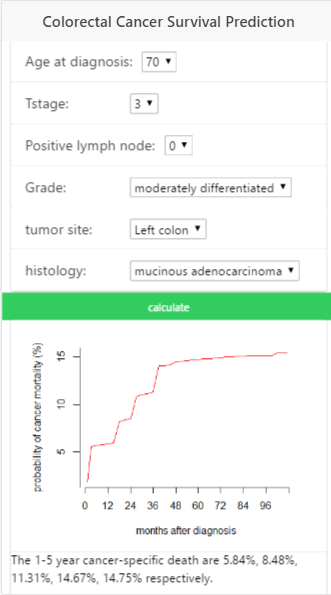


METHODS

**Sub-distribution Analysis of Competing Risks**

The methods we used for estimation, testing and regression modelling of sub-distribution functions in the present of competing risks was performed using cumulative incidence competing risk method, Gray’ test and the Fine and Gray model as described in the references[^1^](#_ENREF_1)^,^[^2^](#_ENREF_2) and implemented in the R package “cmprsk”[^3^](#_ENREF_3)^,^[^4^](#_ENREF_4).

**Random Survival Forests**

In this section, we describe in detail how the random survival forests (RSF) for competing risks were applied to the colorectal cancer dataset. The RSF for competing risks was developed by Ishwaran[^5^](#_ENREF_5), published in the article “Random survival forests for competing risks” and implemented in the R package “randomForestSRC”[^6^](#_ENREF_6).

RSF are extensions of random forests for right censored survival data. A RSF is a collection of randomly grown survival trees. Each tree is grown using an independent bootstrap sample of the learning data using random feature selection at each node. RSF trees are generally grown very deeply with many terminal nodes (the ends of the tree). Trees in competing risk forests are similarly grown, and the estimated values calculated within the terminal nodes to define the ensemble. The procedures used to build a RSF for competing risks were as follows:

1. Draw B bootstrap samples from the learning data.
2. Grow a competing risk tree for each bootstrap sample. At each node of

the tree, randomly select M (M < p) candidate variables. The node is split using the candidate variable that maximizes a competing risk splitting rule.

1. Grow the tree to full size under the constraint that a terminal node should

have no less than n > 0 unique cases.

1. Calculate the cumulative incidence function (CIF), cumulative event-

specific hazard function, and mortality for every event in each tree and event-free survival in each tree.

1. Take the average of each estimator over the B trees to obtain its

ensemble.

The cause-specific hazard function for event j given covariates x is

$$\alpha_{j}\left( t | x \right)=\lim_{\Delta t\to0} \frac{P\{t\leq T^{0}\leq t+\Delta t, \delta^{0}=j|T^{0}\geq t,x\}}{\Delta t}=\frac{f_{j}(t|x)}{S(t|x)}$$

Here, $T^{0}$ represents the event time for the *i*th subject, i=1, …, n, $\delta^{0}$ represents the event type, $\delta_{i}^{0}\in\left\{ 1, \ldots, J \right\}$, where $J\geq1$. $S\left( t | x \right)=P\{T^{0}\geq t|x\}$ is the event-free survival probability function given x.

The probability of an event is determined using the CIF, defined as the probability of experiencing an event of type j by time t; i.e. $F_{j}\left( t | x \right)=P\left\{ T^{0}\leq t, \delta^{0}=j | x \right\}.$ The CIF for event j given covariates x is

$$F_{j}\left( t | x \right)=\int_{0}^{t} \exp\left( -\int_{0}^{S} \sum_{l=1}^{J} \alpha_{l}\left( u | x \right)du \right)\alpha_{j}\left( s | x \right)ds.$$

The expected number of years lost before timeτ is

$$L\left( \tau| x \right)=\tau-\int_{0}^{\tau} S\left( t | x \right)dt=\int_{0}^{\tau} \sum_{l=1}^{J} F_{l}\left( t | x \right)dt.$$

The cause-j mortality is

$$M_{j}\left( \tau| x \right)=\int_{0}^{\tau} F_{j}\left( t | x \right)dt.$$

Let $t1 <t2 <\cdot\cdot\cdot<tm$ denote the $m\leq n$ distinct and ordered event times from ${(T_{i})}_{1\leq i\leq n}$. Let $d_{j}\left( t_{k} \right)=\sum_{i=1}^{n} I(T_{i}=t_{k}, \delta_{i}=j)$ be the number of type j events at $t_{k}$, and $N_{j}\left( t \right)=\sum_{i=1}^{n} I(T_{i}\leq t, \delta_{i}=j)$ be the number of type j events in [0, $t_{k}$]. Define $d\left( t_{k} \right)=\sum_{j} \delta_{j}(t_{k})$, the total number of events occurring at time

$t_{k}$, $N\left( t \right)=\sum_{j} N_{j}(t)$, the total number of events occurring in [0, t], and $Y\left( t \right)=\sum_{i=1}^{n} I(T_{i}\geq t)$, the number of individuals at risk just prior to t. The Nelson-Aalen estimator for the cumulative event-specific hazard function $H_{j}\left( t \right)=E_{x}(\int_{0}^{t} \alpha_{j}(s|x)ds)$ is given by

$$\hat{H}_{j}\left( t \right)=\int_{0}^{t} \frac{dN_{j}(s)}{Y(s)}=\sum_{k=1}^{m(t)} \frac{d_{j}(t_{k})}{Y(t_{k})}$$

Where $m(t)=max\{k:t_{k}\leq t\}$. The Kaplan-Meier estimator for the event-free survival function is given by

$$\hat{S}\left( t \right)=\prod_{s\leq t} \left( 1-\frac{N\left( ds \right)}{Y\left( s \right)} \right)=\prod_{k=1}^{m(t)} (1-\frac{d(t_{k})}{Y(t_{k})}).$$

We use the Aalen-Johansen estimator to estimate $F_{j}\left( t \right):$

$$\hat{F_{j}}\left( t \right)=\int_{0}^{t} \hat{S}\left( u- \right)d\hat{H_{j}}\left( u \right)=\int_{0}^{t} \hat{S}\left( u- \right)Y\left( u \right)^{-1}N_{j}\left( du \right)=\sum_{k=1}^{m(t)} \hat{S}(t_{k}-1)Y\left( t_{k} \right)^{-1}d_{j}(t_{k}).$$

The cause-j mortality is estimated by $\hat{M_{j}}\left( \tau\right)=\int_{0}^{\tau} \hat{F_{j}}\left( t \right)dt.$ We set$\tau$ to be the largest observed time $t_{m}$.

Let ${(T_{i},\delta_{i},x_{i})}_{1\leq i\leq n}$ denote the learning data. As stated earlier, a RSF tree is grown using an independent bootstrap sample of the learning data. Let $c_{i,b}$ be the number of times case i occurs in bootstrap sample b. To define the CIF for the *b*th tree, take a case’s covariate x and drop it down the tree. Let $h_{b}(x)$ denote the indices for cases from the learning data whose covariates share the terminal node with x. Denoting node-specific event counts by $N_{j,b}\left( t | x \right)=\sum_{i\in h_{b}(x)} c_{i,b}I\{T_{i}\leq t,\delta_{i}=j\}$ and the number at risk by $Y_{b}\left( t | x \right)=\sum_{i\in h_{b}(x)} c_{i,b}I\{T_{i}\geq t\}$, we define x’s CIF as

$$\hat{F}_{j,b}\left( t | x \right)=\int_{0}^{t} \hat{S}_{b}(u-|x)Y_{b}\left( u | x \right)^{-1}N_{j,b}(du|x),$$

Where $\hat{S}_{b}\left( t | x \right)=\prod_{u\leq t} (1-\sum_{j} N_{j,b}\left( du | x \right)/Y_{b}(u|x))$ is x’s Kaplan-Meier estimate of event-free survival. The ensemble estimates of the CIF and the cause-j mortality, respectively, equal

$$\bar{F}_{j}\left( t | x \right)=\frac{1}{B}\sum_{b=1}^{B} \hat{F}_{j,b}(t|x),$$

$$\bar{M}_{j}\left( \tau| x \right)=\int_{0}^{\tau} \bar{F}_{j}\left( t | x \right)dt:=\frac{1}{B}\sum_{b=1}^{B} \hat{M}_{j,b}\left( \tau| x \right).$$

For our analysis settings, we used the Gray’s test for the splitting rules. Because our purpose is to predict the CIF of events of interest, it is better to use the Gray’s test because it selects variables based on their direct effect on the cumulative incidence. Different numbers of unique cases at terminal nodes, trees and variables were randomly selected as candidates for each node split in these experiments (the results are shown below).We used 30 unique cases for terminal nodes, 300 trees and 2 candidate variables.

During the RSF for competing risks modelling process, out-of-bag (OOB) ensembles were used. For each bootstrap sample, we randomly selected 63% of the records for training and the remaining 37% for validation. Hence, we obtained a cross-validation based C-index according to the principles described in the reference[^5^](#_ENREF_5) and open sourced R codes. For the Fine and Gray model, we used a bootstrapping method to evaluate the mean concordance index (0.7543) and its confidence interval (95% 0.7510-0.7583).


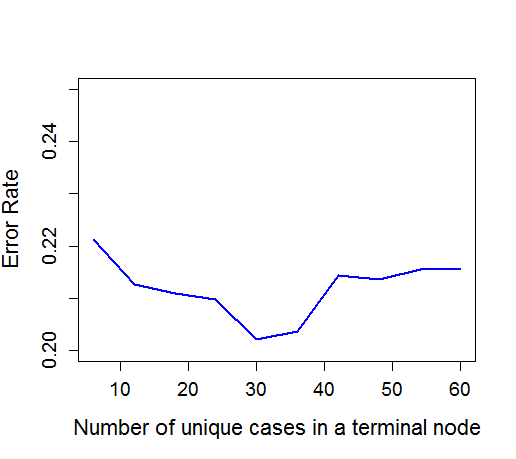


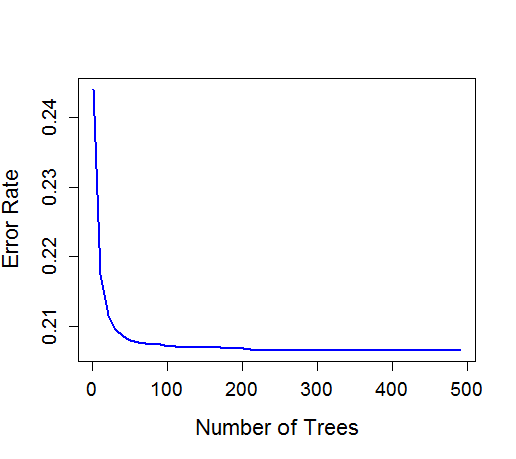


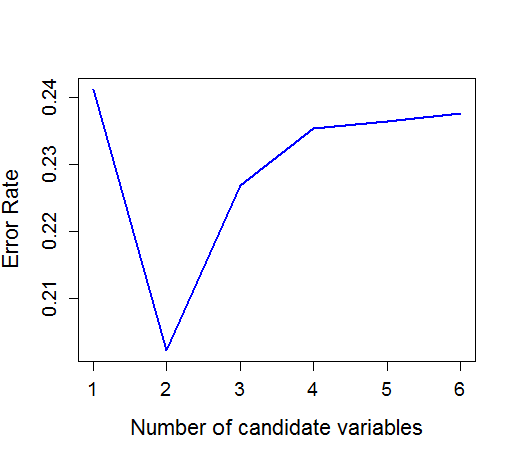


References

1. Gray RJ. A Class of K-Sample Tests for Comparing the Cumulative Incidence of a Competing Risk. *The Annals of Statistics* 1988; **16**(3): 1141-54.

2. Fine JP, Gray RJ. A proportional hazards model for the subdistribution of a competing risk. *J Am Stat Assoc* 1999; **94**(446): 496-509.

3. Gray B (2014). Subdistribution Analysis of Competing Risks. R package version 2.2.7, https://cran.r-project.org/web/packages/cmprsk/index.html. .

4. Therneau T (2015). A Package for Survival Analysis in S. version 2.38, <http://CRAN.R-project.org/package=survival>.

5. Ishwaran H, Gerds TA, Kogalur UB, Moore RD, Gange SJ, Lau BM. Random survival forests for competing risks. *Biostatistics* 2014; **15**(4): 757-73.

6. Ishwaran H. and Kogalur U.B. (2015). Random Forests for Survival, Regression and Classification (RF-SRC), R package version 2.0.5. .
